# Editorial: The impact of primary care on cancer screening program performance: strategies to increase uptake and effectiveness

**DOI:** 10.3389/fmed.2025.1696813

**Published:** 2025-09-22

**Authors:** Cecilia Acuti Martellucci, Enrique Quintero, Christos Lionis

**Affiliations:** ^1^Department of Medical and Surgical Sciences, University of Bologna, Bologna, Italy; ^2^Department of Gastroenterology, Hospital Universitario de Canarias, Instituto Universitario de Tecnologías Biomédicas, Universidad de La Laguna, Tenerife, Spain; ^3^Laboratory of Social and Family Medicine, School of Medicine, Clinic of Social and Family Medicine, University of Crete, Heraklion, Greece; ^4^Department of Psychology, School of Social Sciences and Humanities, University of Limassol, Limassol, Cyprus

**Keywords:** primary care physicians, colorectal cancer, cervical cancer, breast cancer, organized screening, early diagnosis, personalized screening

Cancer screening is recommended in many countries, and is often implemented in the form of free, organized, Public Health interventions, especially in the case of breast, cervical, and colorectal cancer (CRC). Indeed, CRC screening with either fecal immunochemical testing (FIT) or colonoscopy—targeting women and men equally—results in similarly significant reductions in both CRC-related incidence and mortality (1, 2). However, the uptake of screening varies greatly across countries and even smaller regions. CRC screening is an extreme example: participation remains suboptimal in several countries, in average-risk and in familial-risk populations (3–5). In recent years, a study from Crete reported an increased incidence of CRC among young adults (< 50 years), in a population with historically low incidence (6). It is fundamental to investigate uptake as the effectiveness of screening depends, among other factors, on a high participation by the target population (7). In addition, changes in the epidemiology of several preventable cancers highlight the importance of early intervention in primary care. For example, while the incidence of breast cancer is slowly rising in two European regions (Östergötland, Sweden, and Crete, Greece), mortality has increased in Crete compared with Sweden (8). Several studies suggest that Primary Care Physicians, or General Practitioners (GPs), have a substantial influence on the screening adherence of their assisted subjects' (9–12), as counseling by GPs has been associated to higher participation (11). Yet, thus far, interventions targeting GPs have rarely been tested in order to improve the uptake and appropriateness of cancer screening (13–16). The present Research Topic aimed to collect and highlight quality evidence on the impact of GPs on the performance of screening programmes using, for instance, risk-stratification or other organizational changes.

The work by Petrik et al. provides insights on a multi-component strategy employed by primary care clinics (PCCs) to increase participation to FIT, in the rural areas of Oregon, United States. In this study, the clinics adhering to the intervention adopted a strategy including posting of FIT kits, and training and support to medical assistants, who then navigated the patients resulting positive, through the phone. Higher FIT return and CRC screening rates were more likely in clinics which were able to mail an introductory letter, had experience in CRC screening, and attended the health plan-clinic meetings. Similarly, Kruse-Diehr et al. pilot-tested a method to increase participation to CRC screening in Appalachian Kentucky, finding that the great majority of individuals returned a FIT when it was provided in combination with an exploratory “talking card.” These approaches, although dependant on the organization of each PCC, are promising for countries such as the United States and Australia (17, 18), where remoteness is a much greater issue than in Europe (19, 20).

Similarly, research on cervical cancer screening also verified the impact of a strategy to improve uptake, although in the setting of opportunistic screening in Catalonia. Peremiquel-Trillas et al. distributed HPV self-sampling kits through pharmacies (upon SMS invitation), finding a participation rate of 80%. Self-sampling was already shown to improve participation (21), and Catalonia is set to implement it within its population-based programme. Gezimu et al., instead, conducted a narrative review of the perception of cervical screening by female healthcare professionals. Most of the examined studies reported poor knowledge, unfavorable attitudes, and low uptake, but also suboptimal service accessibility, and lack of training. If confirmed, these findings call for improved screening access and training of providers.

Concerning risk-based screening programmes, research is still ongoing on their effectiveness and feasibility (22). Some algorithms are long-established, as is the case for breast cancer (23), for which personalized screening schedules are being tested in RCTs (22), aiming to reduce not only the incidence of advanced cancers, but also the overall tests and procedures (24). Guan et al., in a qualitative study set in Georgia, conducted interviews among PCC professionals, to assess their attitudes toward genetic risk-based breast screening, and observed that the only obstacles to intensifying screening tests in high-risk women were the limited knowledge and unclear referral protocols, while performing fewer tests in low-risk women was less acceptable.

Moving away from conventionally recommended screening, two papers explored the opportunity to screen for melanoma, a rarer but rapidly growing malignancy (25). The intervention tested by Becker et al. was an educational campaign, including online and on-site training, developed to promote an effective skin examination, and disseminated throughout PCCs in Oregon. Over two thousand primary care providers participated to at least one training component, corresponding to about one quarter of those contacted, and the campaign is still ongoing. Further, the study by Pillai et al. proposes a deep-learning algorithm, which reached accuracy, in identifying the malignant nature and the diagnostic category, both above 90%, suggesting that similar tools could become a precious aid within primary care.

More in general, Jeong et al. investigated whether changes in demography correspond to changes in the participation to screening programmes, in Korea. Indeed, decreases in the size of the population were associated with lower participation to cancer screening, for a reduction of about 10%. In a country where out-of-pocket accounts for a substantial part of the health expenditure (26), the elderly groups remaining in depopulated regions are likely unappealing to PCCs (27). Their findings underscore the importance of promptly adapting primary care to specific demographic patterns, and to implement care pathways which integrate services from primary to tertiary hospitals (26).

Finally, Jerjes et al. warn against the underestimation of cancer risk in younger patients. A rise in CRC incidence in young adults was recently reported in the literature (6), and, while differential diagnosis justifiably takes cancer in little account for young patients, GPs should not entirely disregard it. A constant update on the epidemiological trends and appropriate diagnostic procedures is recommended, as well as the introduction of standardized digital decision-support tools, which may aid professionals in the timely identification of malignancies (28).

Despite the evidence linking advice by GPs to cancer screening uptake, studies involving primary care providers and targeted at improving the effectiveness of cancer screening programmes are still scarce. Future efforts should be directed at performing pragmatic experimental research, investigating both effectiveness and financial sustainability. The evidence that this Research Topic conveys could facilitate the design of the future work.
